# Comparing fracture resistance of CAD/CAM polyetherketoneketone ″PEKK″, carbon reinforced polyetheretherketone ″CR PEEK″ and nano-zirconia implant abutments receiving digitally milled nano-zirconia and glass ceramic crowns: an in vitro study

**DOI:** 10.1186/s12903-026-09150-4

**Published:** 2026-08-03

**Authors:** Mohamed Atif Elkholy, Hoda A. Fansa, Ahmed Sameh Abd El Shakour, Hoda Gaafar Hassan Hammad

**Affiliations:** 1 Prosthodontic Department, Faculty of Dentistry, Shibīn al Kawm City, Menoufia University, Menoufia, Egypt; 2 Faculty of Dentistry, Al -Ahliyya Amman University, Amman City, Jordan; 3 Faculty of Dentistry, Alexandria University, Alexandria, Egypt; 4 Prosthodontic Department, National Research Centre, Cairo, Egypt; 5 Prosthodontic Department, Misr University for Science and Technology, Cairo, Egypt

**Keywords:** Carbon-Reinforced Polyetheretherketone, CAD/CAM polyetheretherketone, polyetherketoneketone, Crown, Nano-zirconia, Glass Ceramic, Monolithic lithium disilicate, Implant Abutments, Fracture Resistance

## Abstract

**Background **The presented in vitro study aimed to evaluate the fracture resistance of implant-supported systems with different combinations of abutment–crown materials, namely: Computer-Aided Design/ Computer-Aided Manufacturing ″CAD/CAM″ Nano-zirconia ″NZr″, carbon reinforced polyetheretherketone ″CR PEEK″ and polyetherketoneketone ″PEKK″ dental implant abutments were coronally restored by digitally milled monolithic lithium disilicate ″MLD″ as a type of glass-ceramic ″GC″ and Nano-zirconia ″NZr″ crowns following a thermomechanical aging protocol.

**Materials and methods** A total of 60; Nano-zirconia ″NZr″, carbon reinforced polyetheretherketone ″CR PEEK″, and polyetherketoneketone ″PEKK″ maxillary premolar abutments with titanium base were distributed into three equal groups (*n* = 20). Each group was divided according to the fabricated and cemented CAD/CAM milled crown into two equal subgroups: Nano-zirconia ″NZr″ and monolithic lithium disilicate ″MLD″ (*n* = 10). The ceramic crowns were surface treated and cemented to the abutments by applying the recommended adhesive resin cement. Those study specimens underwent thermo-mechanical aging (49 N, 1.2 × 10^6^ cycles, 5–55 °C). Afterward, the fracture resistance test was performed, and the statistical analyses were made for all subgroups using two-way (ANOVA) analysis of variance and the Tukey post hoc tests (*α* = 0.05).

**Results** Nano-zirconia implant abutments cemented to NZr crowns exhibited the highest fracture resistance values of all subgroups. In addition, ″CR PEEK″ implant abutments reported higher fracture resistance values than those of ″PEKK″. Within each implant abutment group ″NZr″ crowns demonstrated significant high fracture resistance values over their ″MLD″ counterparts (*p* < 0.0001).

**Conclusions** Considering limitations of the presented in vitro study, after thermomechanical aging, recently introduced ″NZr″ implant abutments exhibited higher fracture resistance than ″CR PEEK″ that again reported higher fracture resistance than ″PEKK″ abutments. Generally, all abutment materials might withstand the physiologic occlusal forces. Information about the long-term survival of these recently studied implant abutments types seems limited.

**Supplementary Information:**

The online version contains supplementary material available at 10.1186/s12903-026-09150-4.

## Introduction

Achieving long-term clinical success of the implant-abutment-crown assembly relies largely on stability, biomechanical integrity and aesthetics complex, particularly under functional and parafunctional occlusal loads. Consequently, adequate selection of abutment and crown materials having competent fracture resistance and favorable aesthetic properties, especially in posterior regions, is usually the main clinical concern for contemporary implant dentistry [1–3].

The wonderful evolution in nanotechnology, CAD/CAM technology and dental implants has significantly improved restoring patients to their normal aesthetics, function, speech, comfort, and health. A proper decision for the prosthetic materials, on the other hand, is considered a critical factor for clinical stability and serviceability of implant-supported restorations [4–7].

High crystallization of zirconia (ZrO_2_; zirconium dioxide) increases stability of its chemical and mechanical characteristics, including resistance to low-temperature degradation and corrosion, flexural strength, fracture resistance, micro-hardness, fracture toughness, and radiopacity. Specifically, polycrystalline ZrO_2_ has three different crystallographic structures: cubic (high translucency), monoclinic, or tetragonal (high strength) systems that transform from one phase into another, induced by combined stimuli of humidity, temperature, and stress. In biomedical applications, they typically exist as metastable tetragonal partially stabilized zirconia ″PSZ″; where at room temperatures, the energy is still trapped within the ZrO_2_ ceramic material, preventing the crystal system from transforming from the tetragonal to the monoclinic phase ″Tetragonal to Monoclinic Transformation; TMT″ [8–10]. Afterward, Yttria-stabilized Tetragonal Zirconia Polycrystals ″Y-TZP″ were generated with different compositions of yttria ″Yttrium oxide; Y_2_O_3_″ percentage (3,4,5 mol% of yttria) to enhance the stability of ZrO_2_ phases [9, 10]. Notwithstanding, fatigue in the metastable 3Y-TZP structure creates a stress-induced transformation at the end of the generated micro-cracks that propagate, and only the particles nearby transform from the tetragonal to the monoclinic phase; this characteristic ZrO_2_ process is called ″Transformation Toughening of Zirconia″ ″TT″ [10–12].

Consequently, ″NZr″ is recently innovated to provide a natural white esthetic color, excellent biocompatibility, high fracture toughness, which determine it as a structurally effective high-performance ceramic material for the construction of bridges, crowns, inserts, abutments, and implants [11, 12].

Classified as a glass-ceramic; ″LS_2_″, was initially composed of 65 vol% small needle-shaped crystals (3–6 μm × 0.8 μm) dispersed in a glass amorphous matrix [13, 14] Commercially, ″LS_2_″ material was available as ingots for use according to the ″Heat-Pressing″ fabrication procedure; simulating the classic ″Lost Wax Technique″. Aiming for an appealing reproduction of optical natural teeth characteristics, constructed LS_2_ cores were lately veneered with a very translucent fluorapatite ceramic, containing 19–23% of fluorapatite crystals (Ca_5_(PO_4_)_3_F) embedded in a glassy matrix [15, 16].

″PAEK″ is a semi-crystalline thermoplastic high-performance polymer ″HPP″ family characterized by high mechanical strength, toughness, high-temperature stability, and resistance to hydrolytic degradation. In addition, both ″PEEK″ [with its modifications; ″ceramic-reinforced and carbon-fiber reinforced ″CR PEEK″] and ″PEKK″ are ″HPP″ derived from ″PAEK″[17]. These technologically modified polymer biomaterials demonstrate elastic moduli ″Young’s moduli″ approximating that of the human cortical bone that contribute to improved stress distribution and shock-absorbing behavior at the implant-abutment interface. Interestingly, these ″HPP″ are used for the construction of healing caps, implants, abutments, crowns, and framework material in dental prostheses [18, 19].

Interestingly, the increased ketone group in its chemical structure offers PEKK superior biocompatibility, better shock absorbing capacity, compressive strength, and a relatively higher melting temperature compared to unreinforced ″PEEK″ [20, 21]. However, ″PEKK″ has a compressive strength simulating that of dentin, it has a lower elastic modulus than that of dentine. Furthermore, the possibility of fabricating ″PEKK″ materials with various degrees of crystallinity, produces ″PEKK″ formulations with different grades of hardness. Accordingly, ″PEKK″ is used as post-core, implant, and framework material in fixed and removable prostheses [22, 23].

Despite the widespread clinical implementation of ″PEKK″, ″CR PEEK″, and ″NZr″ abutments, the available comparative data regarding their fracture strength when restored with digitally milled ″NZr″ and ″GC″ crowns are still limited [24, 25]. Therefore, the study aimed to comparatively evaluate fracture resistance of ″CAD/CAM″ fabricated ″PEKK″, carbon-fiber reinforced ″CR PEEK″, and nano-zirconia implant abutments that were coronally restored with digitally milled ″NZr″ and ″MLD″ crowns; which was a crucial issue for optimizing material selection in high-load implant-supported prostheses. The first null hypothesis suggested that there would be no observed significant difference in fracture resistance among the three investigated implant abutment materials. The second null hypothesis suggested that there would be no observed significant difference in fracture resistance between the two crown materials used and no observed interaction between the implant abutment and crown materials.

## Materials and methods

### Ethical aspects

The research protocol was approved by the Medical Research Ethical Committee ″MREC″ of National Research Center ″NRC″; Federal Wide Assurance No: FWA 14,747, approval for exempt review No: EX-03430426.

### Sample size calculation

A priori power analysis was conducted using G*Power (version 3.1.9.7) to determine the minimum sample size required for a two-way ANOVA (2 × 3 design). The calculation was based on data extracted from a previous study [26]. The utilized parameters were as followed: effect size (Cohen’s f) = 0.423, α error = 0.05, and power level = 0.80. The analysis indicated that a total sample size of 58, yielding *n* = 10 samples per group, would be enough to detect significant main effects and interactions among the study groups.

### Materials

## Methods

### Study designing and grouping

**Fig. 1:**
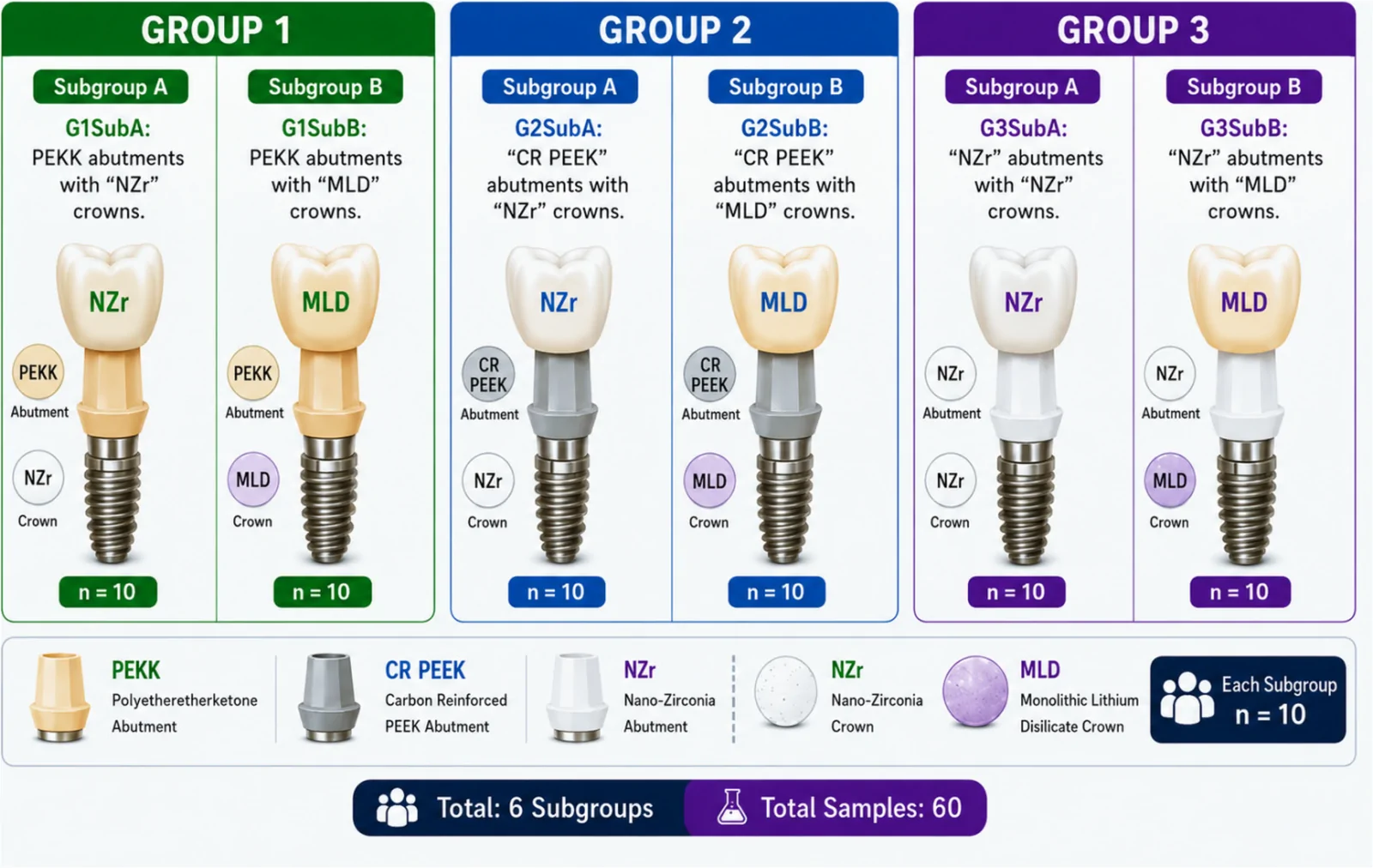
Schematic diagram for grouping of the studied implant abutments and crowns

The 60 implant analogues with their attached Ti-abutment bases were distributed into three equal implant abutment groups (*n* = 20): Group1 was CAD/CAM ″PEKK″, Group 2 was ″CR PEEK″, and Group 3 was ″NZr″; (Table 1). In addition, each implant abutment group was evenly distributed to two equal crown subgroups (n = 10): subgroup A was ″NZr″ and subgroup B was ″MLD″ as a glass ceramic″. (Table 1; Figure 1).

**Table 1:** Grouping of investigated implant abutments and crowns

Group 1	Subgroup A	G1SubA: PEKK abutments with ″NZr″ crowns.
	Subgroup B	G1SubB: PEKK abutments with ″MLD″ crowns.
Group 2	Subgroup A	G2SubA: ″CR PEEK″ abutments with ″NZr″ crowns.
	Subgroup B	G2SubB: ″CR PEEK″ abutments with ″MLD″ crowns.
Group 3	Subgroup A	G3SubA: ″NZr″ abutments with ″NZr″ crowns.
	Subgroup B	G3SubB: ″NZr″ abutments with ″MLD″ crowns.

### Study setting

A total of 60 implant analogues (3.8 × 10 mm; simulating the average of the implant dimensions suitable for the maxillary premolar area) were individually inserted in cylindrical autocured resin molds in a perpendicular direction. Young’s modulus of that set mold material was (~ 1.47–2.3) GPa [27] approximating that of the human trabecular bone (~ 0.2-5 GPa) [28].

Meanwhile, a standardized device for proper paralleling (Dremel^®^ Moto-Tool Model 395, WI, USA) was used in order to maintain the implant analogues in the accurate positions till the resin completely set.

**Fig. 2:**
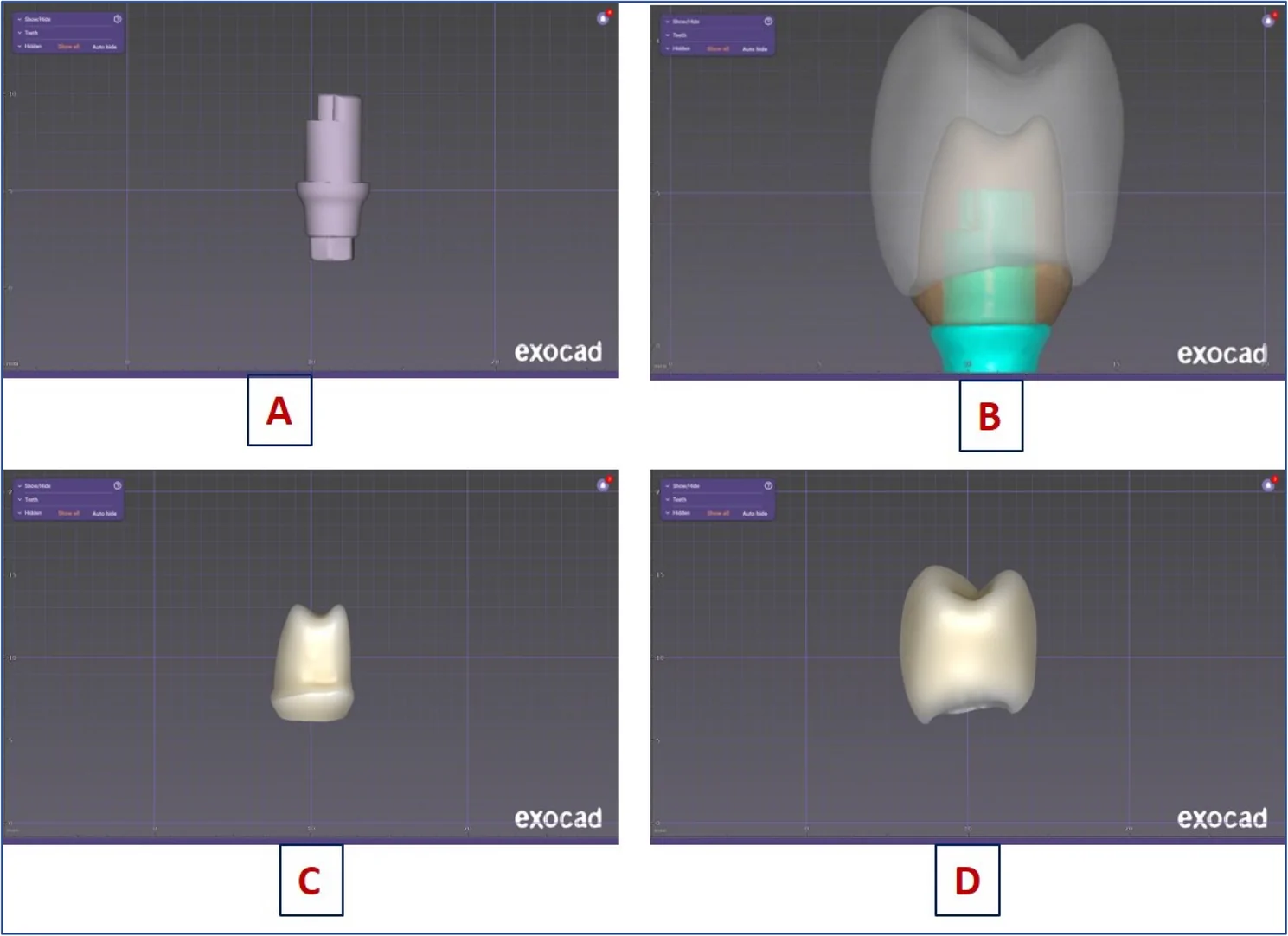
CAD Exocad software for designing implant abutments and crowns. **A** Ti-base dimensions. **B** Ti-base with implant abutment and crown. **C** Implant abutment dimensions. **D** Crown dimensions

Moreover, a Ti-abutment base with a platform switching configuration (characterized by: 0.5 mm shoulder finishing line, 3 mm width, and 4.5 mm height) was manually screwed to each study implant analogue (Fig. 2; A). Afterward, each Ti-base abutment screw access channel was properly sealed with wax. A desktop scanner (Medit i900 Mobility (i900M), Seongbuk-gu, Seoul, Korea) was utilized to scan the Ti-bases, and a computer-aided design ″CAD″ software (Exocad Elefsina, Version 3.2, Darmstadt, Germany) was applied to precisely design the implants’ abutments and crowns.

In addition, all implant abutments and permanent ceramic crowns were digitally designed with ″CAD″ software and were manufactured following a standardized ″CAD/CAM″ workflow (Fig. 2; B). The scanning of the specimens was performed using a laboratory structured-light scanner (InEos X5; Dentsply Sirona, Bensheim, Germany; accuracy ± 5 μm). Afterward; processing of those ″CAD″ designs were performed using hyperDENT ″CAM″ software (Version 5.2; Technology Group, Munich, Germany), where a 50 μm uniform cement space was set.

The implant abutment width was 5.2 mm buccolingually and 5.8 mm mesiodistally, while the height was 7 mm; (Fig. 2; C). The digitally milled crown width was 8 mm mesiodistally and 8.5 mm buccolingually, and its height was 9 mm (Fig. 2; D) [27]. Then, those STL designing files were sent from ″CAD″ software to the ″CAM″ software. Accordingly; a five- axis milling machine (Glidewell Dental Labs, VHF Cam-facture AG, Ammerbuch, Germany) was used for the construction of the implant abutments and their respective crowns.

The study specimens were equally divided into three main groups (*n* = 20) depending on the dental implant abutment biomaterial i.e. CAD/CAM ″PEKK″, ″CR PEEK″ and ″NZr″; (Table 2). Therefore, each group was again divided into two equal subgroups (*n* = 10) based on the milled crown biomaterial into ″NZr″ and monolithic lithium disilicate ″MLD″ as a type of glass ceramic ″GC″. The lithium disilicate crystals contain 70% fine grains of ″Li_2_Si_2_O_5_″ that are embedded in a glassy matrix (Table 2). Furthermore; the Ti-base abutment components were air-abraded with Al_2_O_3_ (50 μm particle size) for 20 s at 2 bar pressure, then washed for 10 min in an ultrasonic bath that contained 80% ethanol. Subsequently, the Ti-bases were dried, and a thin layer of Ceramic Bond was applied for 60 s to react; (Table 2) [29].

**Table 2:** Materials of Implant abutments, crowns and adhesive systems

Material Name	Manufacturer	Classification	Primary Composition / Active Ingredients
Dental Implant Analogue	Powerbone (Manisa, Türkiye)	Lab Component	Stainless Steel or Titanium alloy (Grade 5)
Dental implant Ti-Base	Powerbone (Manisa, Türkiye)	Prosthetic Base	Titanium Alloy (Ti-6Al-4 V)
Pekkton^®^ ivory	Cendres+Métaux Milano, Italia.	HPP (Polymer)	Polyetherketoneketone (PEKK)
CR PEEK Discs	Invibio Biomaterial Solutions, Invibio Ltd., Lancashire, United Kingdom.	Reinforced Polymer	Polyetheretherketone (PEEK) + 30% Carbon Fibers
IPS e.max CAD	Ivoclar Vivadent	Glass Ceramic	70% fine-grain lithium disilicate crystals (Li_2_Si_2_O_5_), which are embedded in a glassy matrix.
Nano Zirconia Discs	Harvest Dental	Nanozirconia Ceramic	Advanced 4Y zirconia, multi-layered with (1000–1200 MPA) composed of yttria-stabilized tetragonal zirconia polycrystal (Y-TZP) comprising: ZrO_2_ + HFO_2_ +Y_2_O_3,_ Y_2_O_3,_ HFO_2,_ Al_2_O_3_ Other Oxides.
Technovit 4000	Heraeus Kulzer, Germany	self-cured acrylic resin	Modified Methyl Methacrylate (MMA)
Anaxblend Metal Bonder	Anaxdent	Metal Primer	MMA with phosphoric acid monomers
Anaxblend PEKK Bond	Anaxdent	Adhesive System	Methyl Methacrylate / MMA-based primer
Ceramic Bond	VOCO GmbH, Germany	Silane Primer	3-Methacryloxypropyltrimethoxysilane (MPS)
MKZ Primer	Bredent GmbH, Germany	Universal Primer	Acetone, 10-MDP Monomer
Visio.link	Bredent GmbH	Resin Primer	MMA, PETIA, Photo initiators
Monobond Plus	Ivoclar Vivadent	Universal Primer	Silane, 10-MDP, and Disulfide acrylate
Clearfil Ceramic Primer	Kuraray Noritake	Universal Primer	Silane coupling agent and 10-MDP
DTK Adhesive	Bredent Medical	Dual cure composite Cement	Bis-GMA based composite resin
Multilink Hybrid abutment cement	Ivoclar Vivadent	Luting Composite	Dimethacrylates, Barium glass fillers
Panavia V5	Kuraray Noritake	Dual cure resin Cement	Bis-GMA, TEGDMA, 10-MDP

Group 1: Subgroup A (GISubA; PEKK abutments with ″NZr″ crowns). Concerning G1A subgroup, ten PEKK blanks were used to construct the CAD/CAM ″PEKK″ implant abutments. Each Ti base was properly screwed into its implant analogue. The G1A subgroup was for ″PEKK″ abutments that were produced using Harvest Nano-Zirconia Discs. The Ti bases and ″PEKK″ abutments were sandblasted using 110 μm Al_2_O_3_ particles at 3 bars for the Ti base and 2 bars for the ″PEKK″ abutments. Then, for the cementation procedures; a metal bond was distributed on the outer surfaces of the Ti bases and a ″PEKK″ bond was filling the intaglio surfaces of the abutments that rested on the Ti bases. Furthermore; Multilink Hybrid Abutment cement was used to lute both Ti bases and ″PEKK″ abutments (Table 2). All manipulation procedures were performed in compliance with the received recommendations of PEKK’s manufacturer for bonding the Ti-base.

Group 1: Subgroup B (GISubB; ″PEKK″ abutments with ″MLD″ crowns). About G1B; Ti bases were also luted to ″PEKK″ abutments using Multilink Hybrid Abutment cement (Table 2). Furthermore, all procedures were accomplished in accordance with the PEKK’s manufacturer recommendations of the Ti-base bonding as GIA subgroup [30].

Group 2: Subgroup A (G2SubA; ″CR PEEK″ abutments with ″NZr″ crowns). An additional MKZ primer was applied to cement the ″CR PEEK″ abutments to the Ti bases while following the manufacturer’s MKZ instructions. Regarding ″CR PEEK″ abutments, the implemented adhesion protocol included; initial surface material abrasion using 110 μm Al_2_O_3_ at 3 bars, followed by application of the Visio.link primer, then luting to Ti bases with DTK as an adhesive resin cement, and light cure for 90s (Table 2) [31, 32].

Group 2: Subgroup B (G2SubB; ″CR PEEK″ abutments with ″MLD″ crowns). Cementation of ″CR PEEK″ abutments to Ti-bases were performed as G2SubA subgroup; (Table 2) [33].

Group 3: Subgroup A (G3SubA; ″NZr″ abutments with ″NZr″ crowns). The G3A subgroup was for esthetic hybrid zirconia abutments, which were produced from Harvest Nano-Zirconia Discs, and their flexural strength exceeds 1,200 MPa. Those ″NZr″ discs are advanced 4-yttria-stabilized tetragonal zirconia polycrystal ″4Y-TZP″ composed of: ZrO_2_ + HFO_2_ +Y_2_O_3_, Y_2_O_3_, HFO_2_, Al_2_O_3_ and other oxides [34]. Therefore; sandblasting with 110 μm Al_2_O_3_ was done at 2 bars pressure for the inner surfaces of ″CAD/CAM″ milled ″NZr″ abutments and 3 bars for the outer surface of the Ti-bases as per the manufacturer’s recommendations to achieve optimum cementation. Afterward, MKZ primer was applied to those sandblasted surfaces for 30s, then air dried, and finally luted with DTK adhesive dual-cure composite cement; (Table 2).

**Fig. 3:**
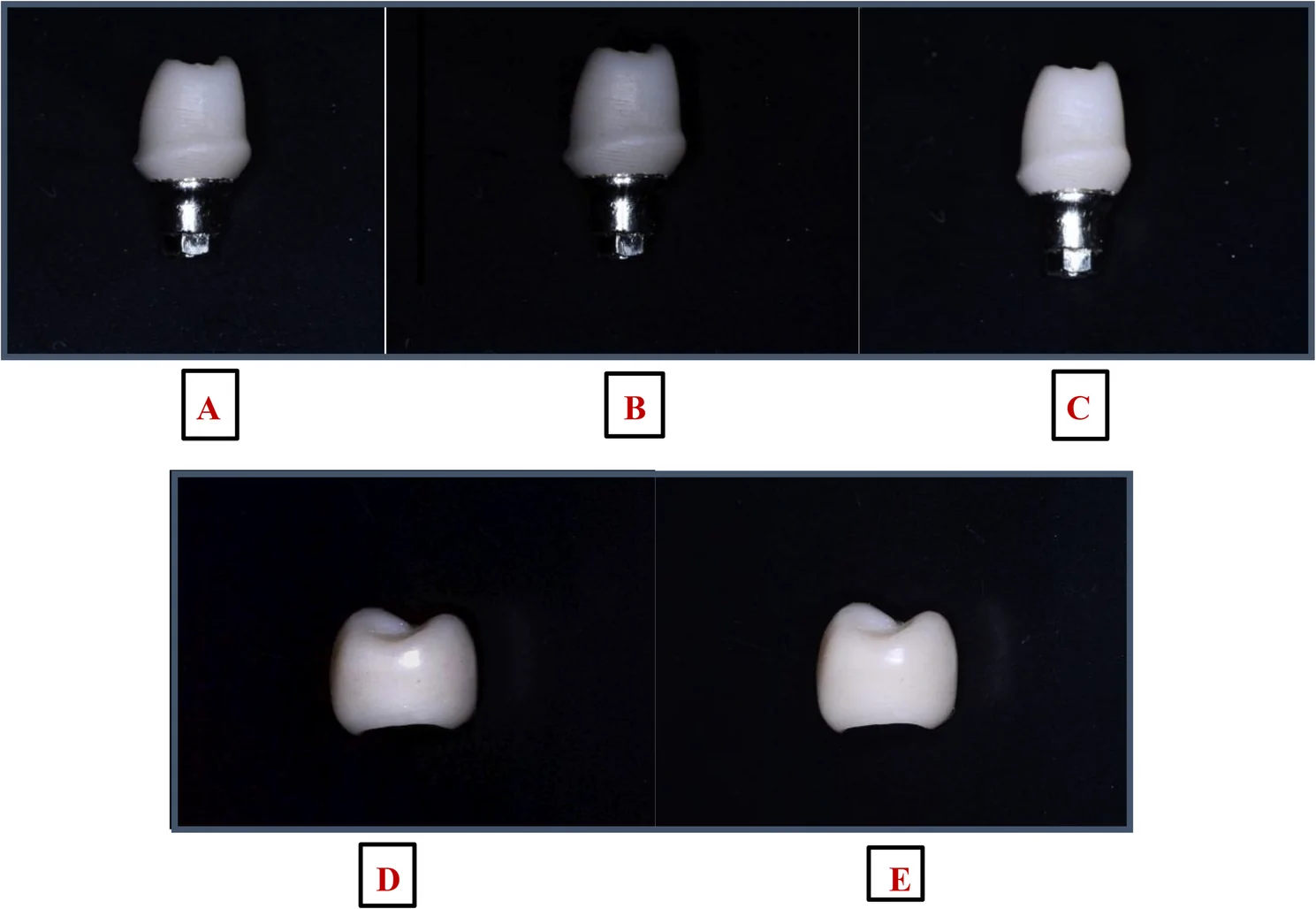
Implant abutments and crowns. **A** CAD/CAM PEKK abutment. **B** Carbon reinforced ″CR PEEK″ abutment. **C** Nano-zirconia abutment. **D** Nano-zirconia crown **E** Monolithic lithium disilicate crown

Group 3: Subgroup B (G3SubB; ″NZr″ abutments with ″MLD″ crowns). Bonding of ″NZr″ abutments to the Ti-bases was attained with dual-cure DTK adhesive composite cement. When polymerization of the resin had been completed, all the study abutments (Fig. 3; A, B&C) were firmly screwed to their corresponding analogs at a 25 Ncm torque. Again, they were re-screwed 10 min later in order to prevent any loss of the preload. Then, those screw holes were carefully sealed using Teflon tape and a flowable resin composite (Table 2).

CAD/CAM precise software was designed to construct all the study crowns with the same dimensions (i.e., 9 mm height, 8 mm mesiodistal width, and 8.5 mm buccolingual width). Therefore; 60 crowns were fabricated for all study groups that were divided into: 30 ″NZr″ crowns that were milled from CAD/CAM ″NZr″ blocks, then sintered, and finished and 30 partially crystalline ″MLD″ crowns that were milled and subsequently fully crystallized in a ceramic furnace to fit the definitive maxillary premolar abutments [35, 36], (Fig. 3; D&E) Consequently, no preparation was attempted, aiming to avoid any probable weakening of that ceramic material in the prefabricated implant abutments.

#### Crowns’ surface treatment and cementation protocols

Surface treatment of those ceramic crowns’ intaglio surfaces was performed as followed: The ″NZr″ crowns were duly sandblasted using 50 μm Al_2_O_3_ at a 10 mm distance and 2 bars pressure for 30 s, then a universal primer (Monobond Plus) was implemented for 60 s. On the other hand; ″MLD″ crowns were etched using 9.5% hydrofluoric acid only for 30 s, rinsed during 60 s, and finally air-dried. Afterward; a Clearfil acid primer plus was applied for 30 s, then air dried.

**Fig. 4:**
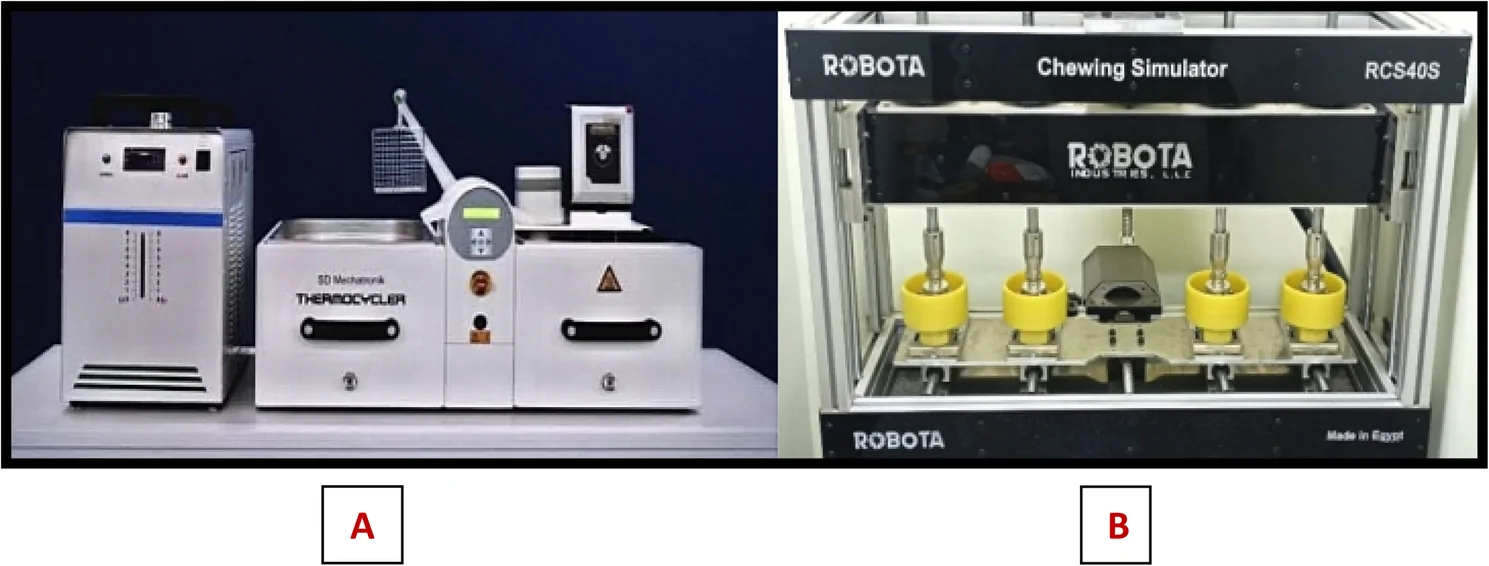
**A** Thermocycling machine, **B** Chewing simulator machine

In addition, the study abutment surfaces were properly cleaned and treated in compliance with each material-specific protocol. Under a standardized seating pressure, the crowns were adhered to their abutments with a dual-cure Panavia V5 resin cement (Fig. 4; A) [37, 38]. After complete cement setting, the excess material was removed, and each specimen was profoundly light-cured (Elipar S10, 3 M ESPE) from each aspect for exactly 30 s. Then, each cemented specimen was stored distinctly at 37 °C for 24 h till the thermomechanical cyclic loading test.

#### Thermocycling and chewing simulation

All specimens were subjected to thermo-mechanical aging in a dual-axis 6-chamber chewing simulator (49 N load, 1.6 Hz frequency, 5 °C to 55 °C temperature, 1.2 × 10^6^ cycles, dwelling time 120 s) that should correspond to 5 years of clinical performance [17]. Jointly, a stainless-steel ball (6 mm diameter) was adjusted to simulate the concave surface of the crowns as an antagonist during the dynamic cyclic loading (Fig. 4; A).

#### Fracture resistance testing

**Fig. 5:**
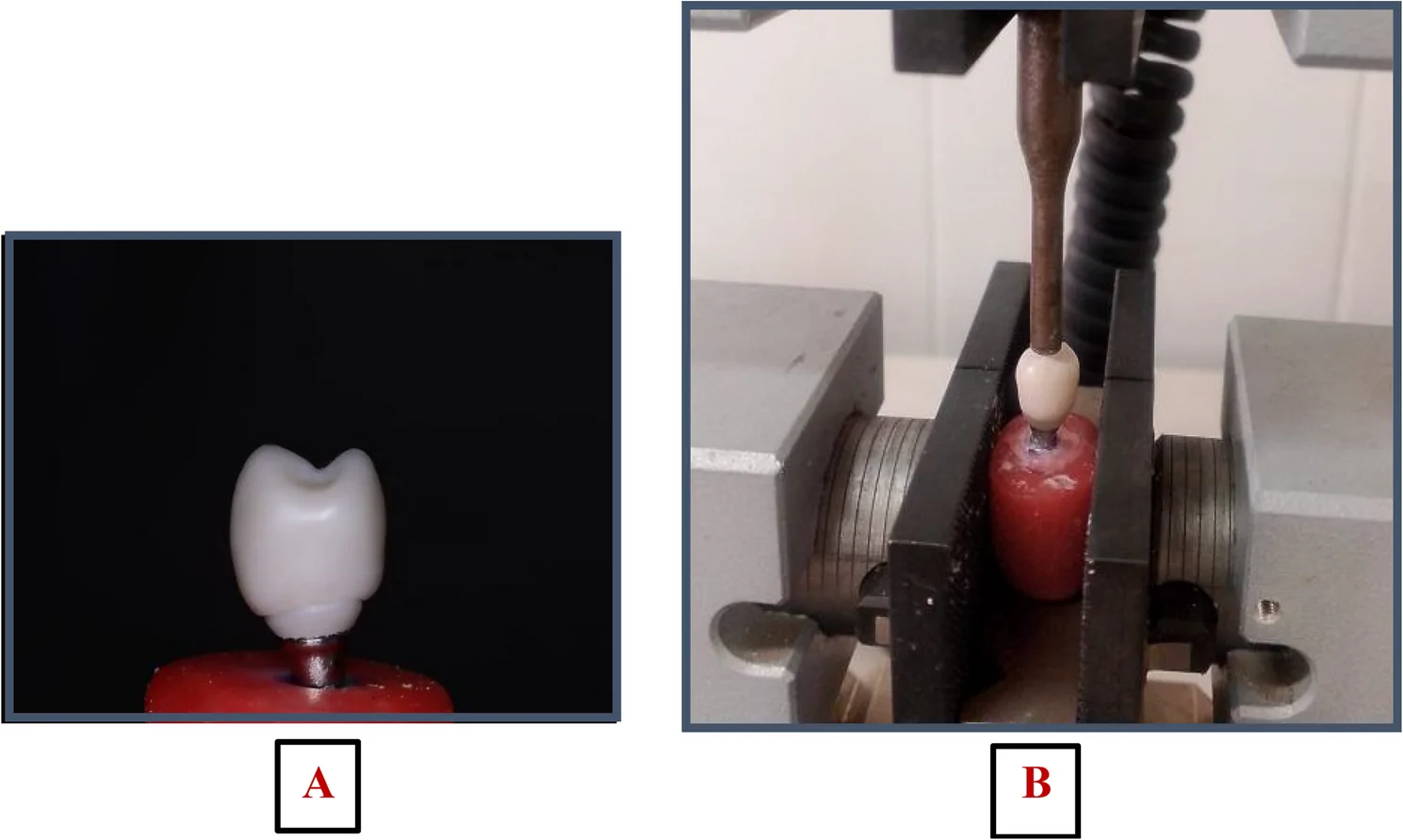
Fracture resistance test using a universal testing machine. **A** An implant ti-base with the constructed abutment and crown. **B** Failure of a study specimen

Each subgroup specimen was mounted to a universal testing machine (Model 3345; Instron Industrial Products, Norwood, MA, USA) at its lower compartment, where a static increasing compressive load was applied till its complete failure (Fig. 5; B). A stainless-steel ball (5 Kn load cell, and 6 mm diameter) loading piston was used to apply the force vertically toward the occlusal surface till the complete fracture. Then, all the obtained fracture load readings in Newton were reported by specific computer software (Instron Bluehill Lite Software) [39].

#### Statistical Analysis

The statistical analysis of the study was done with the GraphPad Prism software program, version 8.0.1, 2018, Inc., CA, USA. All reported data was tested for normality employing the D’Agostino and Pearson omnibus normality statistic test. Obtained data was expressed as means and confidence interval. Afterward, a two-way ANOVA prospered by Tukey’s post-hoc test was implemented for determining the probable statistical difference among the investigated subgroups. Failure mode distributions across material subgroups were compared using chi-square contingency table analysis (6 subgroups × 3 failure types). Statistical significance was considered at *p* values < 0.05.

## Results

At the end of thermocycling and chewing simulation tests, all study specimens were properly checked, and they were free from any visible deformation, any screw fracture, or any loosening of the crown or screw. After aging, the determined survival rate of all the subgroup specimens was 100%.

**Fig. 6:**
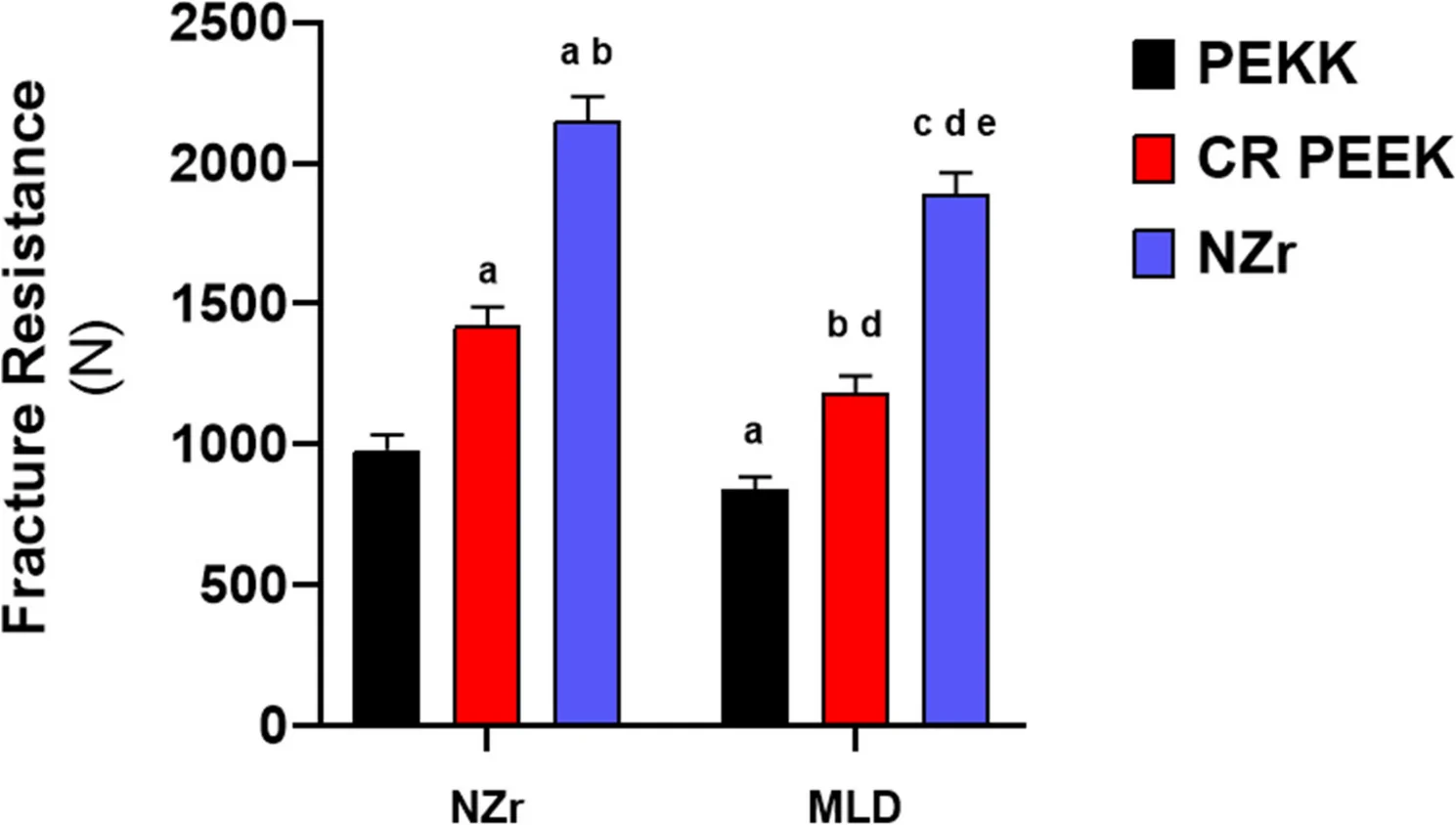
Fracture resistance values “N” of all the study implant abutments/crowns subgroups. Data was presented as mean [95% confidence interval] (*n* = 10). ^a^ *p* < 0.05 vs. the G1SubA (PEKK/NZr); ^#^ *p* < 0.05 vs. the G2SubA (CR PEEK/ NZr); ^c^ *p* < 0.05 vs. the G3SubA (NZr / NZr); ^d^ *p* < 0.05 vs. the G1SubB (PEKK/ MLD group); ^**e**^ *p* < 0.05 vs. the G2SubB (CR PEEK/ MLD)

Obtained study data was presented as means (*n* = 10), as shown in (Tables 3 and 4; Fig. 6). Two-way ANOVA revealed that the abutment material had a profound impact on the fracture resistance, exhibiting a remarkably large effect magnitude (F _(2, 54)_ = 736.5, *p* < 0.0001, η_p_² = 0.965). The crown material also demonstrated a highly significant large effect (F _(1, 54)_ = 79.28, *p* < 0.0001, η_p_² = 0.595). Conversely, the interaction between crown and abutment types was not statistically significant with a negligible effect magnitude (F _(2, 54)_ = 2.400, *p* = 0.1003, η_p_² = 0.082).

**Table 3:** Fracture resistance values (in Newton “N”) of the different study subgroups

Crown Abutment	PEKK	CR PEEK	NZr
NZr	980 [932.5–1027.5]	1420 [1359.8–1480.2]^a^	2150 [2073.9–2226.1]^a, b^
MLD	840 [800.7–879.3]^a^	1180 [1124.2–1235.8]^b, d^	1890 [1823.5–1956.5]^c, d, e^

Data was presented as mean [95% confidence interval] (*n* = 10)

^a^*p* < 0.05 vs. the G1SubA (PEKK/NZr)

^b^*p* < 0.05 vs. the G2SubA (CR PEEK/ NZr)

^c^*p* < 0.05 vs. the G3SubA (NZr / NZr)

^d^*p* < 0.05 vs. the G1SubB (PEKK/ MLD group)

^**e**^*p* < 0.05 vs. the G2SubB (CR PEEK/ MLD)

**Table 4:** Two-way ANOVA summary for fracture resistance of tested groups

Source of Variation	Sum of Squares (SS)	Degrees of Freedom (DF)	Mean Square (MS)	F-value (DFn, DFd)	*P*-value	Partial Eta Squared (ηp²)
Abutment	12,684,000	2	6,342,000	F _(2, 54)_ = 736.5	< 0.0001	0.965
Crown	682,667	1	682,667	F _(1, 54)_ = 79.28	< 0.0001	0.595
Interaction	41,333	2	20,667	F _(2, 54)_ = 2.400	0.1003	0.082
Residual (Error)	464,967	54	8,611	-	-	-

Applied Tukey’s multiple comparisons revealed a clear hierarchy in load-bearing capacity, where ″NZr″ abutments yielded the highest fracture resistance mean values (exceeding 2000 N with ″NZr″ crowns), followed by ″CR PEEK″, while ″PEKK″ exhibited the lowest mean values across all implant abutment groups. Consequently; within each abutment category, ″NZr″ crowns subgroups consistently outperformed ″MLD″; for instance, ″NZr″ crowns on ″NZr″ abutments showed a significant mean difference of 260 N over their ″MLD″ counterparts (*p* < 0.0001); (Tables 3 and 4; Fig. 6). Ultimately, the data demonstrated that a ″NZr″-″MLD″ system provided superior fracture resistance, and the choice of a relatively high strength ceramic abutment was far more critical to the system’s ultimate strength than the selection of the crown material itself.

The ″CR PEEK″ abutment with ″NZr″ crown subgroup ″G2SubA″ and the ″NZr″ abutment with ″NZr″ crown subgroup ″G3SubA″showed a significant increase in fracture resistance compared to ″PEKK″ abutment with ″NZr″ crown subgroup ″G1SubA″ (*p* < 0.0001); while ″PEKK″ abutment with ″MLD″ crown subgroup ″G2SubB″ exhibited a significant reduction compared to the same subgroup (*p* < 0.05). In addition, ″NZr″ abutment with ″NZr″ crown subgroup ″G3SubA″ exhibited a significant increase in fracture resistance compared to ″CR PEEK″ abutment with ″NZr″ crown subgroup ″G2SubA″; while ″CR PEEK″ abutment with ″MLD″ crown subgroup ″G2SubB″exhibited a significant reduction in fracture resistance compared to the same group (*p* < 0.0001). Moreover, ″NZr″ abutment with ″MLD″ crown subgroup ″G3SubB″ showed a significant reduction in fracture resistance compared to ″NZr″ abutment with ″NZr″ crown subgroup ″G3SubA″ (*p* < 0.0001). Furthermore, ″CR PEEK″ abutment with ″MLD″ crown ″G2SubB″ and the ″NZr″ abutment with ″MLD″ crown ″G3SubB″ subgroups exhibited a significant increase in fracture resistance compared to those of ″PEKK″ abutment with ″MLD″ crown subgroup ″G1SubB″ (p < 0.0001). ″NZr″ abutment with ″MLD″ crown subgroup ″G3SubB″ showed a significant increase in fracture resistance compared to ″CR PEEK″ abutment with ″MLD″ crown subgroup ″G2SubB″ (p < 0.0001) (Tables 3 and 4; Fig. 6).

### Failure mode analysis

Subsequent to that static fracture resistance test, specimens’ failure modes were individually assessed post-fracture via visual inspection as well as stereomicroscopy (×40 magnification), and failure modes were categorized as followed: Type I: Crown chipping/fracture, Type II: Abutment fracture, Type III: Combined crown-abutment fracture (Table 5).

**Table 5:** Failure mode analysis

Subgroup	Type I: Crown (%)	Type II: Abutment (%)	Type III: Combined (%)
G1SubA (PEKK/NZr)	90	10	0
G1SubB (PEKK/MLD)	100	0	0
G2SubA (CR PEEK/NZr)	30	70	0
G2SubB (CR PEEK/MLD)	40	60	0
G3SubA (NZr/NZr)	20	30	50
G3SubB (NZr/MLD)	30	40	30
Chi-Square (χ^2^) test	χ^2^ = 39.60, df = 10, *p* <0.0001		

Data presented as absolute percentages (%) (*n* = 10 per subgroup)

Regarding the G1″PEKK″ group; failures were primarily located at the crown level. Investigated G1SubA ″PEKK/NZr″ subgroup exhibited Type I failure in nine study specimens (90%), and Type II failure in one specimen (10%); however, Type III failure was not detected in any specimen (0%). Moreover, in G1SubB ″PEKK/MLD″ subgroup, Type I failure was recorded in all specimens (*n* = 10; i.e. 100%), while Type II and Type III failure modes were not observed in any specimens (0%).

For G2 ″CR PEEK″ group; failure patterns were shifted predominantly toward the implant abutment. In the G2SubA ″CR PEEK/NZr″ subgroup; three specimens (30%) were recorded as Type I failure, and seven specimens (70%) were observed as Type II failure, while Type III failure was not found (0%). Furthermore, in G2SubB ″CR PEEK/MLD″ subgroup; Type I failure was noted in four specimens (40%), and Type II failure accounted for six specimens (60%), while; Type III failure did not show (0%).

**Fig. 7:**
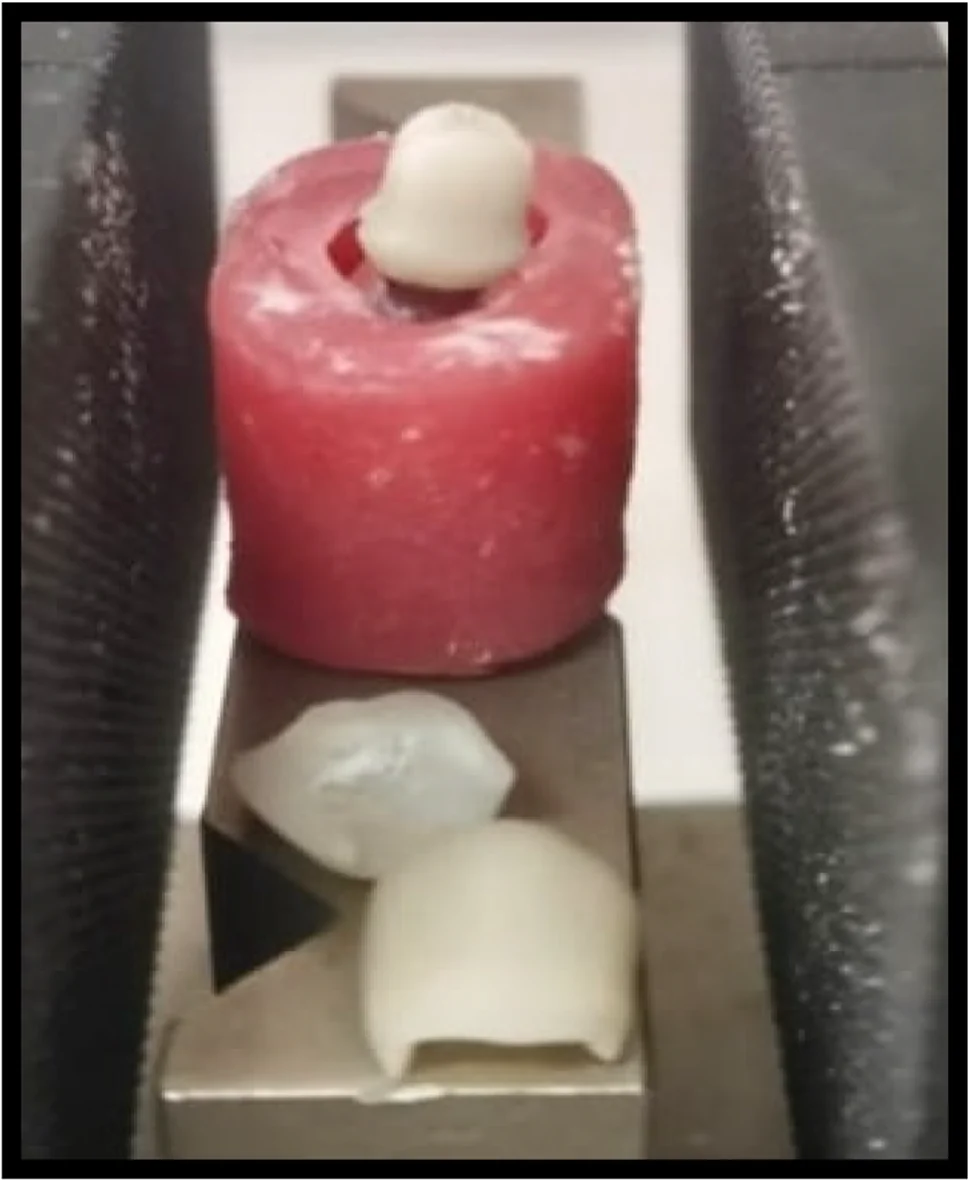
Fractured crown after fracture resistance test

Concerning the G3″NZr″ group; the combined crown-abutment failure mode (Type III) was the most prevalent. In the G3SubA (NZr/NZr) subgroup; Type III (combined crown-abutment failures) was reported in five specimens (50%), Type II (abutment failure) was observed in three specimens (30%), and Type I (crown failure) accounted for only two specimens (20%). In the G3SubB (NZr/MLD) subgroup, Type I was observed in three specimens (30%), Type II was noted in four specimens (40%), and Type III was found in the remaining three specimens (30%) identified as failures. Accordingly; failure mode analysis revealed a highly significant association between material subgroup and failure mode distribution (χ^2^ = 39.60, df = 10, *p* < 0.0001) (Table 5; Fig. 7).

The brittle nature of ″NZr″ ceramic crowns exhibited a catastrophic, unstable rapid crack propagation process with or without little plastic deformation when supported by stiff ″NZr″ abutments, when stresses were efficiently transmitted across a well-bonded interface, and the fracture pattern might spread through the crown into the abutment, producing combined catastrophic failures (Type III). On the other hand, ″NZr″ ceramic crowns supported by the more compliant polymeric ″PEKK″ abutments again failed in a brittle manner with cracks and localized chipping (Type I) because ″PEKK″ substructure absorbed energy, deformed and blunted crack growth. Conversely, ″CR PEEK″ abutments have relatively higher mechanical properties than neat ″PEKK″ due to carbon fiber reinforcement which reduced that protective compliance and shifted peak stresses into the abutment, thus; increasing the risk of abutment fracture rather than isolated ″NZr″ crown chipping (Type II). Therefore, for a crown-adhesive interface-abutment assembly; the crown’s intrinsic brittleness, the elastic moduli mismatch, and the interface bonding quality together determined whether an initiated flaw would remain confined (i.e., repairable chipping) or it would catastrophically propagate through both crown and abutment.

Concerning interface stress distribution for stiff pairings (NZr crown/NZr abutment), stress was efficiently transmitted across the interface, creating a continuous high-stress pathway that facilitated combined failures (Type III). Moreover, with the more compliant abutment or when an energy dissipating layer (e.g. PEKK, resilient cement) existed, the interface might interfere with the crack propagation, acting as a blocker at site of localized failure (debonding, chipping) rather than permitting through the component fracture.

Those findings addressed the clinical implications and repairability of different failure patterns. Type I (crown chipping) was usually managed by chairside repair or by crown reconstruction with preservation of both the abutment and implant. Furthermore; Type II (abutment fracture) was commonly more invasive and costly, as it required abutment replacement and mostly necessitated access to the implant connection. Unfortunately; Type III (combined catastrophic failure) subjected implant to risk of damaging loads; which might compromise implant components, and typically required surgical removal and replacement.

## Discussion

In the present in vitro study, a Ti-base with a platform switching known as; ″platform shifting″ configuration was used as a modern designed dental implant prosthetic solution to enhance the soft tissue stability and preserve the crestal bone by employing an abutment of a smaller diameter than that of the implant platform. The applied design aimed to clinically create an inward “horizontal step” at the implant-abutment interface, therefore, displacing the microgap away from the marginal bone and consequently, reducing stresses on the crestal bone region. That implemented platform shift was also in agreement with Mohamed LM, et al.; 2024 [40] who applied that implant Ti-based platform switch to minimize the typical marginal bone resorption that might occur following the implant insertion. Consequently, the inflammatory cell infiltrate that is often produced around the abutment-implant connection, is compelled away from the adjacent crestal bone [41]. In addition, thermo-mechanical aging that was implemented for the study specimens was convenient with Schäfer T, et al.; 2024 [17].

However, obtained fracture resistance results revealed a significant main effect of abutment material (F _(2, 54)_ = 736.5, *p* < 0.0001) and a significant main effect of crown material (F _(1, 54)_ = 79.28, p < 0.0001); it did not show any significant interaction between those two study factors (F _(2, 54)_ = 2.4, p = 0.1003) (Table 3; Fig. 6).

Statistically, the G3 ″NZr″ implant abutment group showed the highest fracture resistance values, followed by those for G2 ″CR PEEK″, then those for G1 ″PEKK″; where all reported high fracture resistance values (Table 3; Fig. 6). Concerning obtained ″NZr″ material findings, they might be attributed to the nanostructure of ZrO_2_ that increases the elastic modulus and mechanical performance, combined with the yttria modified crystalline structure that offers resistance to ″low temperature degradation″; “Aging of ZrO_2_, Tetragonal to Monoclinic Phase Transformation”, [33, 42]“ceramic corrosion” or ″Hydrothermal Degradation″, and the transformation toughening ″TT″ mechanism of zirconia, which all contribute to increasing its fracture resistance, providing an admired polish ability, and reducing the abrasive wear potential on opposing arch teeth [43, 44]. This ″TT″ strengthens the ″ZrO_2_″ material by two different mechanisms: first, by dissipating the energy causing fracture during the transformation and second; through residual compressive stresses [45]. Those arguments agreed with other studies [42–44].

Accordingly, advanced multi-layered 4Y-TZP is a recent 4th-generation ″ZrO_2_″, that is engineered to attain high compressive strength (1000–1200 MPa) with good translucency; addressing limitations of earlier, opaquer 3Y-TZP and relatively less durable 5Y-PSZ materials. The composition of multi-layered 4Y-TZP includes ZrO_2_, hafnium oxide, Y_2_O_3_ (~ 4 mol% for tetragonal and cubic crystal blend), and a small amount of alumina for aging resistance. This multi-layered material establishes superior aesthetics compared to 3Y-TZP and greater strength than 5Y-PSZ, making it suitable for monolithic crowns, multi-unit bridges, and implant-supported restorations [8, 10].

Despite ″NZr″ producing admirable, highly promising aesthetic and mechanical characteristics in many in vitro studies across different applications of dentistry, [43–46] more longitudinal clinical studies are still required before this technology can be unequivocally adopted in evidence-based practice.

The reported G2 ″CR PEEK″ implant abutment higher fracture resistance values compared to those of G1″PEKK″ might be assigned to the addition of 30% carbon fibers to the biocompatible ″PEEK″. That incorporation significantly increases the dimensional stability, toughness, hardness, compressive strength (simulating that of dentine), flexural strength, and fracture resistance of ″PEEK″. Furthermore, those findings might be because of the different surface treatments implemented in each group and the various adhesive bonding systems applied with each material. However, the ″CR PEEK″ material’s grayish and dull appearance restricts its monolithic use for posterior crowns and requires veneering for esthetic purposes. Those predictions were convenient with Alqurashi H, et al.; 2021 [47] and Ramaswamy K, et al.; 2021 [48]. Furthermore, ″CR PEEK″ is a biocompatible, thermoplastic, semi-crystalline high-performance polymer derived from ″PAEK″ family [48, 49].

Concerning G1 ″PEKK″ implant abutment fracture resistance values, they might be acceptable compared to those of human bone. Inherently, ″PEKK″ material has the highest biocompatibility of all ″PAEK″ polymers [37] and it has a high content of ketone groups that contributes to improved stress distribution, toughness, elastic modulus, higher resistance at elevated temperatures, and shock-absorbing behavior at the implant-abutment interface [49, 50]. Those findings agreed with Zol SM, et al.; 2023 [32]. Indeed, information on the long-term survival of ″PEEK″ and ″PEKK″ as implant abutment material is still limited in the literature.

In addition, ″NZr″ crown material showed markedly higher fracture resistance values than those of ″MLD″; which is characterized by high esthetic attributes due to its 19–23% content of fluorapatite crystals (Ca5(PO_4_)3 F) embedded in a glassy matrix, as was confirmed by Kandil MT, et al.; 2025 [15] and Vallerini BD, et al.; 2024 [51]. Commercially, ″LS_2_″; known as IPS e.max Press, is a high-strength GC composed of 65 vol% needle-shaped crystals in a glass matrix. It offers superior mechanical properties, including a flexural strength of 370–460 MPa and fracture toughness of 2.8–3.5 MPa [15, 51].

Therefore, the null hypothesis was rejected while comparing the three studied implant abutment materials, ″PEKK, CR PEEK, NZr″ as well as the two crown materials ″NZr, MLD″. While it was accepted for assessed interaction between the ″abutment and crown″ as two study factors. Thus, suggesting that implant abutment performance was consistent regardless the overlying crown biomaterial. That obtained feedback was congruent with similar research findings [47, 52].

Interestingly, that in vitro study performed thermomechanical aging followed by static load-to-fracture testing. Despite these procedures providing useful information, it didn’t answer whether the implant restorations would survive progressive fatigue failure.

Unfortunately, visual and spectroscopic assessments for failure mode were limited and should be investigated in future research (Table 5; Fig. 7). Acknowledged study limitations included lack of fatigue survival analysis, crack initiation analysis, step-stress fatigue testing, survival probability assessment, or Weibull analysis. These tests are important because they simulate the cyclic occlusal forces and aging conditions encountered clinically, allowing prediction of restoration durability, reliability, and probability of failure over time. Fatigue-to-failure and step-stress protocols help to identify crack propagation behavior and the endurance limit of restorative materials under repeated loading, while Weibull analysis provides information about material reliability and structural consistency. Interestingly, for each implant abutment and crown material used in the study, various surface treatments and different adhesive bonding systems were implemented following the manufacturer’s instructions, which presented other variables that might affect the obtained results and might be considered limitations. Consequently, future research designed in vitro as well as in vivo research will be essentially needed before encouraging ″NZr, PEEK or PEKK″ as alternatives implant abutment biomaterials for clinically relevant outcomes.

## Conclusions

Based on the obtained findings in this limited in vitro study, the following can be concluded:

1. These findings suggest that implant abutment material plays an important role in the mechanical performance of the implant–abutment–crown assembly; however, further in vivo and long‑term studies are needed to confirm these results.
2. Within the limitations of this in vitro study, CAD/CAM and Nano-zirconia technologies seem promising to improve the fracture resistance and esthetic outcomes of the implant-supported prostheses.
3. Further in vitro investigations and clinical studies are recommended to assess the long-term performance and serviceability of ″NZr, CR PEEK and PEKK″ as recent tentative alternatives for posterior implant restorations.

## Supplementary Information

Supplementary Material 1.

Supplementary Material 2.

## Data Availability

No datasets were generated or analysed during the current study.
