# Supplementary material for: Comparing fracture resistance of CAD/CAM polyetherketoneketone ″PEKK″, carbon reinforced polyetheretherketone ″CR PEEK″ and nano-zirconia implant abutments receiving digitally milled nano-zirconia and glass ceramic crowns: an in vitro study
Source: BMC Oral Health. 2026 Aug 3;26:1545. doi: 10.1186/s12903-026-09150-4 (PMC13505118; doi:10.1186/s12903-026-09150-4)
Supplement: Supplementary file 1 [file 12903_2026_9150_MOESM1_ESM.pdf]

Cover Letter

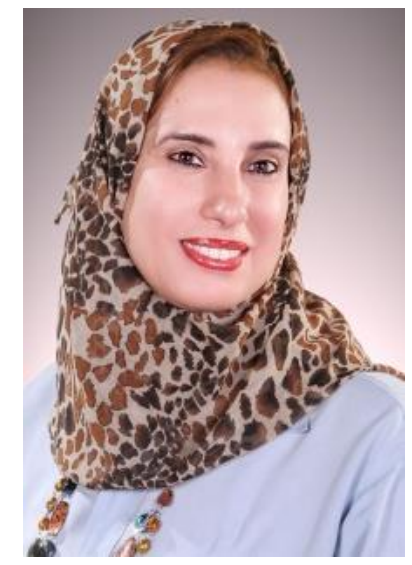

Respected editorial member for **BMC Oral Health Journal**,

Greetings,

Kindly find my updated CV for your reference. I am Egyptian ***Associate professor*** of ***Dental Biomaterials***. I got ***PhD*** in Dental Materials from the collaboration between ***Cairo University*** & ***Rennes 1 University***, Egypt in 2014, ***Master's*** degree in Restorative Dentistry, Cairo University, Egypt in 2001 and ***BDS***, Cairo University in 1994.

I have academic work experience in Universities; **Menoufia University**, ***MUST***, ***GMU*** and ***BMC***.

Please, I want to submit the manuscript: **"Comparing fracture resistance of CAD/CAM Polyetherketoneketone ″PEKK″, carbon reinforced Polyetheretherketone ″CR PEEK″ and nano-zirconia implant abutments receiving digitally milled nano-zirconia and glass ceramic crowns: An in vitro study"** to the BMC Oral Health Journal.

The study is dealing about an important issue in digital dentistry concerning the association of recent different milled implant abutments cemented to varying crown materials on implant analogues as an in vitro study. Accordingly, the fracture resistance was assessed, and the fracture mode was evaluated in the tested groups. That study was performed to investigate the optimum fracture resistance that might be achieved from digital technology of milled crowns and implant abutments.
