# Supplementary material for: Comparing fracture resistance of CAD/CAM polyetherketoneketone ″PEKK″, carbon reinforced polyetheretherketone ″CR PEEK″ and nano-zirconia implant abutments receiving digitally milled nano-zirconia and glass ceramic crowns: an in vitro study
Source: BMC Oral Health. 2026 Aug 3;26:1545. doi: 10.1186/s12903-026-09150-4 (PMC13505118; doi:10.1186/s12903-026-09150-4)
Supplement: Supplementary file 2 [file 12903_2026_9150_MOESM2_ESM.docx]

***Tables and Figures for Implant BMC manuscript 23-6-2026***

***Submission ID: 472bebd5-e03e-4af5-9284-4ddc6bcfef72***

***Deadline: 02 Jul 2026***

***Submission version: v.5.0***

***Title of the manuscript:***

***"Comparing fracture resistance of CAD/CAM Polyetherketoneketone ″PEKK″, carbon reinforced Polyetheretherketone ″CR PEEK″ and nano- zirconia implant abutments receiving digitally milled nano-zirconia and glass ceramic crowns: An in vitro study"***

***Tables***

***Table 1: Materials of Implant abutments, crowns and adhesive systems.***

| Material Name | Manufacturer | Classification | Primary Composition / Active Ingredients |
| --- | --- | --- | --- |
| **Dental Implant Analogue** | Powerbone (Manisa, Türkiye) | Lab Component | Stainless Steel or Titanium alloy (Grade 5) |
| **Dental implant Ti-Base** | Powerbone (Manisa, Türkiye) | Prosthetic Base | Titanium Alloy (Ti-6Al-4V) |
| **Pekkton® ivory** | Cendres+Métaux  Milano, Italia. | HPP (Polymer) | Polyetherketoneketone (PEKK) |
| **CR PEEK Discs** | Invibio Biomaterial Solutions, Invibio Ltd., Lancashire, United Kingdom. | Reinforced Polymer | Polyetheretherketone (PEEK) + 30% Carbon Fibers |
| **IPS e.max CAD** | Ivoclar Vivadent | Glass Ceramic | 70% fine-grain lithium disilicate crystals  (Li_2_Si_2_O_5_), which are embedded in a glassy matrix. |
| **Nano Zirconia Discs** | Harvest Dental | Nanozirconia Ceramic | Advanced 4Y zirconia, multi-layered with (1000-1200 MPA) composed of yttria-stabilized tetragonal zirconia polycrystal (Y-TZP) comprising: ZrO_2_ + HFO_2_ +Y_2_O_3,_ Y_2_O_3,_ HFO_2,_ Al_2_O_3_ Other Oxides. |
| **Technovit 4000** | Heraeus Kulzer, Germany | self-cured acrylic resin | Modified Methyl Methacrylate (MMA) |
| **Anaxblend Metal Bonder** | Anaxdent | Metal Primer | MMA with phosphoric acid monomers |
| **Anaxblend PEKK Bond** | Anaxdent | Adhesive System | Methyl Methacrylate / MMA-based primer |
| **Ceramic Bond** | VOCO GmbH, Germany | Silane Primer | 3-Methacryloxypropyltrimethoxysilane (MPS) |
| **MKZ Primer** | Bredent GmbH, Germany | Universal Primer | Acetone, 10-MDP Monomer |
| **Visio.link** | Bredent GmbH | Resin Primer | MMA, PETIA, Photo initiators |
| **Monobond Plus** | Ivoclar Vivadent | Universal Primer | Silane, 10-MDP, and Disulfide acrylate |
| **Clearfil Ceramic Primer** | Kuraray Noritake | Universal Primer | Silane coupling agent and 10-MDP |
| **DTK Adhesive** | Bredent Medical | Dual cure composite Cement | Bis-GMA based composite resin |
| **Multilink Hybrid abutment cement** | Ivoclar Vivadent | Luting Composite | Dimethacrylates, Barium glass fillers |
| **Panavia V5** | Kuraray Noritake | Dual cure resin Cement | Bis-GMA, TEGDMA, 10-MDP |

***Table 2: Grouping of investigated implant abutments and crowns.***

| **Group 1** | **Subgroup A** | **G1SubA:** PEKK abutments with ″**NZr″** crowns. |
| --- | --- | --- |
|  | **Subgroup B** | **G1SubB:** PEKK abutments with **″MLD″** crowns. |
| **Group 2** | **Subgroup A** | **G2SubA: ″CR PEEK″** abutments with ″**NZr″** crowns. |
|  | **Subgroup B** | **G2SubB: ″CR PEEK″** abutments with ″**MLD″** crowns. |
| **Group 3** | **Subgroup A** | **G3SubA:** ″**NZr″** abutments with ″**NZr″** crowns. |
|  | **Subgroup B** | **G3SubB:** ″**NZr″** abutments with ″**MLD″** crowns. |

***Table 3:*** ***Fracture Resistance Values (in Newton "N") of the Different Study Subgroups.***

| Crown  Abutment | PEKK | CR PEEK | NZr |
| --- | --- | --- | --- |
| NZr | 980 [932.5 - 1027.5] | 1420 [1359.8 - 1480.2] ^a^ | 2150 [2073.9 - 2226.1] ^a b^ |
| MLD | 840 [800.7 - 879.3] ^a^ | 1180 [1124.2 - 1235.8] ^b d^ | 1890 [1823.5 - 1956.5] ^c d e^ |

**Table 4:** **Two-way ANOVA summary for fracture resistance of tested groups.**

| Source of Variation | Sum of Squares (SS) | Degrees of Freedom (DF) | Mean Square (MS) | F-value  (DFn, DFd) | *P*-value | Partial Eta Squared (ηp²) |
| --- | --- | --- | --- | --- | --- | --- |
| Abutment | 12,684,000 | 2 | 6,342,000 | F _(2, 54)_ = 736.5 | < 0.0001 | 0.965 |
| Crown | 682,667 | 1 | 682,667 | F _(1, 54)_ = 79.28 | < 0.0001 | 0.595 |
| Interaction | 41,333 | 2 | 20,667 | F _(2, 54)_ = 2.400 | 0.1003 | 0.082 |
| Residual (Error) | 464,967 | 54 | 8,611 | - | - | - |

***Table 5: Failure Mode Analysis.***

| Subgroup | Type I: Crown (%) | Type II: Abutment (%) | Type III: Combined (%) |
| --- | --- | --- | --- |
| G1SubA (PEKK/NZr) | 90 | 10 | 0 |
| G1SubB (PEKK/MLD) | 100 | 0 | 0 |
| G2SubA (CR PEEK/NZr) | 30 | 70 | 0 |
| G2SubB (CR PEEK/MLD) | 40 | 60 | 0 |
| G3SubA (NZr/NZr) | 20 | 30 | 50 |
| G3SubB (NZr/MLD) | 30 | 40 | 30 |
| Chi-Square (χ^2^) test | χ^2^ = 39.60, df = 10, *p* <0.0001 | | |

***Figures***

***Figure1:*** *Schematic diagram for grouping of the studied implant abutments and crowns.*

***Figure 2:*** *CAD Exocad software for designing implant abutments and crowns.* ***A:*** *Ti-base dimensions.* ***B:*** *Ti-base with implant abutment and crown.* ***C:*** *Implant abutment dimensions.* ***D:*** *Crown dimensions.*

**A	B	C**

**D	E**

***Figure 3:*** *Implant abutments and crowns.* ***A:*** *CAD/CAM PEKK abutment.* ***B:*** *Carbon reinforced ″CR PEEK″ abutment.* ***C:*** *Nano-zirconia abutment.* ***D:*** *Nano-zirconia crown* ***E:*** *Monolithic lithium disilicate crown.*

***Figure 4:*** ***A:*** *Thermocycling machine,* ***B:*** *Chewing Simulator machine.*

**A	B**

***Figure 5:*** *Fracture Resistance Test Using a Universal Testing Machine.* ***A:*** *An implant Ti-base with the constructed abutment and crown.* ***B:*** *Failure of a study specimen.*

***Figure 6:*** *Fracture resistance values "N" of all the study implant abutments/crowns subgroups.*

***Figure 7*:** *Fractured crown after fracture resistance test*.
